# Epigenetics and inheritance of phenotype variation in livestock

**DOI:** 10.1186/s13072-016-0081-5

**Published:** 2016-07-21

**Authors:** Kostas A. Triantaphyllopoulos, Ioannis Ikonomopoulos, Andrew J. Bannister

**Affiliations:** Department of Animal Breeding and Husbandry, Faculty of Animal Science and Aquaculture, School of Agricultural Production, Infrastructure and Environment, Agricultural University of Athens, 75 Iera Odos St., 11855 Athens, Greece; Department of Anatomy and Physiology of Farm Animals, Faculty of Animal Science and Aquaculture, School of Agricultural Production, Infrastructure and Environment, Agricultural University of Athens, 75 Iera Odos St., 11855 Athens, Greece; Wellcome Trust/Cancer Research UK Gurdon Institute, University of Cambridge, Tennis Court Road, Cambridge, CB2 1QN UK

**Keywords:** Epigenetic, DNA methylation, Acetylation, Histone, Transcription, Transgenerational, Inheritance, Imprinting, Nutrition, Livestock

## Abstract

Epigenetic inheritance plays a crucial role in many biological processes, such as gene expression in early embryo development, imprinting and the silencing of transposons. It has recently been established that epigenetic effects can be inherited from one generation to the next. Here, we review examples of epigenetic mechanisms governing animal phenotype and behaviour, and we discuss the importance of these findings in respect to animal studies, and livestock in general. Epigenetic parameters orchestrating transgenerational effects, as well as heritable disorders, and the often-overlooked areas of livestock immunity and stress, are also discussed. We highlight the importance of nutrition and how it is linked to epigenetic alteration. Finally, we describe how our understanding of epigenetics is underpinning the latest cancer research and how this can be translated into directed efforts to improve animal health and welfare.

## Background

The term epigenetics was coined in the 1940s by Conrad Waddington and applied to the possible causal mechanisms acting on the genes that govern phenotypic outcome. Huxley later refined this definition as he realized that the variation in specification of cellular phenotype was not necessarily gene sequence related [1]. Since then, the concept and definition of epigenetics has gradually evolved, slowly diverging from the definition originally prescribed by Waddington [2]. It explains how expression of a gene might be changed and stably maintained by modifications (of DNA and/or histones) without affecting the nucleotide sequence of the gene itself [3]. The ‘memory’ of such activity is transferred between cell generations through mitosis and between organismal generations through meiosis [4].

**Fig. 1:**
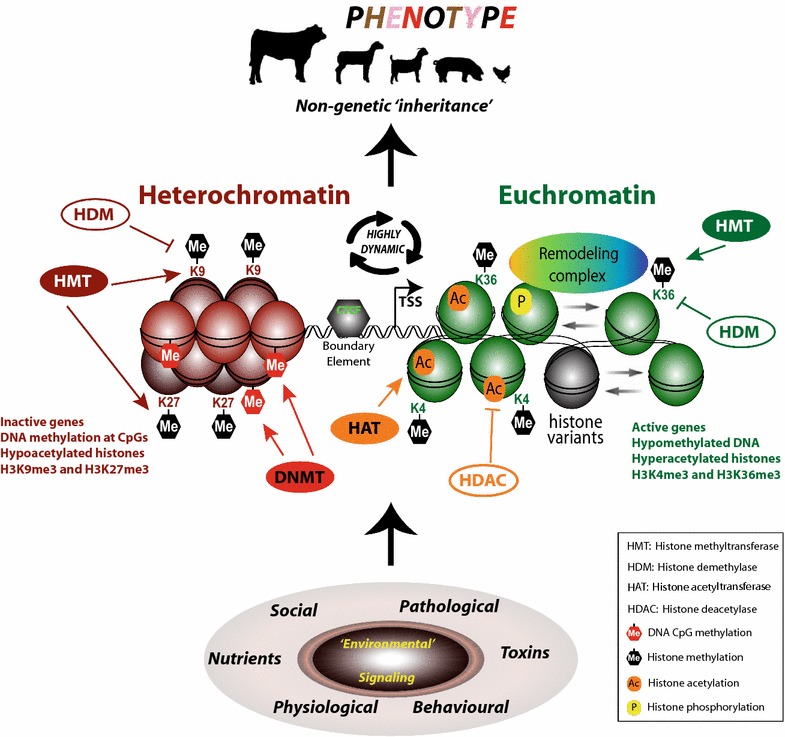
Chromatin modifications and remodelling events in livestock. Different environmental exposures trigger signalling pathways, which affect chromatin structure, thereby affecting gene expression leading to altered phenotypic attributes (phenotype)

Epigenetics has the potential to be very useful in animal breeding, as it may provide information relating to the heritability of complex traits and diseases. In turn this would serve to improve breeding and the genetics of livestock. Indeed, livestock genetics is currently benefiting from massive amounts of genomic information (e.g. arrays that genotype more than 500K SNPs along the bovine genome) that are being incorporated into the prediction of genetic advantage, providing higher accuracy [5] and leading to important changes in the animal breeding industry [6]. However, it is now clear that in addition to DNA sequence information, epigenetic information also determines the overall phenotype (Fig. 1).

Epigenetic processes generate the epigenome and involve DNA methylation, chromatin remodelling, histone modifications, regulation of gene expression by non-coding RNAs, genome instability and any other force that modifies animal phenotype (for review see [7–9]). These processes alter gene expression, and they can affect cell fate and phenotype plasticity as well as behaviour. Various molecular mechanisms are involved, including paramutation, bookmarking, imprinting, gene silencing, transposon silencing, X chromosome inactivation, position effect, reprogramming, transvection and maternal effects [10–20].

Importantly, the relationship between epigenetics and phenotype appears more evident in disease. For instance, aberrant epigenetic pathways have been identified in atherosclerosis [21], osteoarthritis [22], lupus erythematosus [23], imprinting disorders [24], neuropsychiatric disorders [25] and improper gene inactivation in cancer [26]. Epigenome abnormalities related to developmental disorders and late onset adult diseases such as metabolic and mental disorders have also been reported [27]. Epigenetic mechanisms in livestock have mainly focused on the molecular aspects that regulate the expression of certain genes or genomic regions, sometimes as a response to external environmental factors, as described in later sections.

## Epigenetic mechanisms

Epigenetic mechanisms include, but are not limited to, DNA methylation (predominantly at CpG dinucleotides), post-translational modifications (PTMs) of histones, non-coding RNAs (ncRNAs) and chromatin remodelling (Fig. 1).

### DNA methylation

DNA methylation at CpG dinucleotides involves the addition of a methyl group to the 5′ position of the cytosine pyrimidine ring to generate 5-methylcytosine (5^m^C) (Fig. 1). Cytosine methylation also occurs to a lesser extent in non-CpG contexts [28]. Very recently, mammalian genomes have been shown to also possess adenosine methylation, although the physiological consequence of this remains unclear. Nevertheless, modifications involving DNA methylation and alkylation damage of nucleic acids are tightly linked with many diseases [29].

CpG methylation is widespread in mammals and functions directly or indirectly at multiple levels to generally suppress gene transcription. It is also a fundamental mechanism underlying transposon silencing, X chromosome inactivation and gene imprinting [30–35], and at least in part, these effects are due to its link to heterochromatin formation and maintenance. It is performed by DNA methyltransferases (DNMTs) and removed via a pathway involving specific enzymes, for example ten-eleven translocation methylcytosine dioxygenase 1 (TET1), which catalyses conversion of 5^m^C to 5-hydroxymethylcytosine (5^hm^C). This has been proposed as the initial step of active DNA demethylation in mammals [36]. It is well established, that dramatic CpG methylation changes occur during early development [37, 38].

In contrast to CpG methylation in gene promoters, methylation in the body of genes can actually lead to transcriptional activation. Furthermore, differential DNA methylation occurs across distinct cell types [39]. This occurs at developmental and tissue-specific genes as an organism develops. Thus, although the genome is constant, the presence of CpG methylation varies in these genes across cell types [8, 39]. DNA methylation can also be altered at multiple adjacent CpG sites and where this occurs is termed a differentially methylated region (DMR) [40].

### Post-translational histone modifications

DNA in eukaryotic cells is compacted and packaged into a macromolecular complex termed chromatin, the fundamental unit of which is a nucleosome. Nucleosomes consist of a histone protein octamer (2 each of histones H3, H4, H2A and H2B) around which approximately 1.75 turns of DNA are wound. Within this setting, histones are subject to many PTMs that have the potential to encode epigenetic information. Common modifications include acetylation, methylation, phosphorylation and ubiquitylination, and they are deposited on histones, or removed from histones, by specific enzymes (Fig. 1). Importantly, histone modification and DNA methylation pathways are dependent upon each another, and a ‘reinforcing’ cross-talk exists involving interactions between the relevant enzymes and associated factors [7, 41]. Although histone modifications influence transcription, since chromatin is ubiquitous, the modifications also affect all DNA processes including DNA repair, replication and recombination [7].

Chromatin modifications function in two non-mutually exclusive ways. The modifications may directly affect chromatin structure, or they may provide dynamic binding platforms for proteins with specific binding domains. An example of the former is provided by histone acetylation that neutralizes a lysine’s positive charge, thereby disrupting electrostatic interactions. This would facilitate chromatin in adopting a less compact state, consistent with histone acetylation being found at active genes (Fig. 1). Moreover, histone acetyltransferases function as transcriptional coactivators and deacetylases as repressors. A modification that creates a docking site for a protein is exemplified by trimethylation of H3K9 (H3K9me3). This heterochromatic mark is specifically bound by the chromodomain of heterochromatin protein HP1, thereby facilitating the maintenance of heterochromatin [42].

### Non-coding RNA

Although DNA methylation and histone modifications are the most studied epigenetic mechanisms, other epigenetic processes also participate in regulating gene function. An important example is non-coding RNA-mediated regulation of gene expression and chromatin remodelling [35] (Fig. 1). The control and nucleation of sites for epigenetic modification appear to be at least partly mediated by small interfering RNAs and other non-coding RNAs (ncRNAs). Other short ncRNAs, such as piwi RNAs (piRNAs) and microRNAs (miRNAs) have also been implicated in epigenetic inheritance across generations [43, 44].

Long ncRNAs (lncRNAs) regulate DNA processes, such as transcription via *cis*-acting as well as *trans*-acting mechanisms [43]. Although most mechanisms have not been fully elucidated, lncRNAs have been found to act as molecular guides, scaffolds, decoys and allosteric modulators to regulate transcription and chromatin. One mechanism involves the lncRNA forming a triplex structure with specific DNA sequences in gene promoters. At the proto-oncogene *SPHK1*, the lncRNA *Khps1* regulates expression of the gene by forming a triplex DNA/RNA structure within its promoter [45]. This structure serves to recruit histone modifiers to remodel the local chromatin architecture. Given the huge number of lncRNAs within a typical cell, it is certain that these RNAs are going to be immensely important in regulating all DNA processes and they too must be regarded as major players when considering epigenetic mechanisms.

The roles of miRNAs in livestock productivity are beginning to emerge, with miRNAs being shown to be involved in many aspects of farm animal welfare [46], including disease [47], milk production [48] and more specifically adipogenesis [49]. There are now many mature miRNAs identified in cattle (755), sheep (103), pig (306) and chicken (791) that have important functional roles in adipose, skeletal muscle, oocyte and early embryonic development (http://www.mirbase.org) [46]. Future studies will undoubtedly focus on identifying the mRNAs targeted by miRNAs and the physiological processes regulated by the miRNAs.

### Chromatin remodelling

Chromatin remodelling involves the repositioning or restructuring of nucleosomes within chromatin to facilitate or inhibit access to the nearby DNA (Fig. 1). It is predominantly performed by ATP-dependent chromatin remodelling complexes that move, eject or restructure nucleosomes [50, 51]. Dynamic remodelling of chromatin imparts an epigenetic regulatory role in several key biological processes, including egg cell DNA replication and repair, apoptosis, development and pluripotency [50]. However, the dynamics in chromatin organization during development is not a unique system in all vertebrates but differs, for example between mammals (e.g. mouse) and non-mammals (e.g. chicken) [52]. Importantly, aberrant chromatin remodelling has been associated with human diseases, such as cancer [53, 54].

## Challenges in epigenetics for livestock breeding

Recent technological advances in the field of epigenomics include genome-wide next-generation sequencing, dynamic imaging of genomic loci, quantitative proteomics and computational analyses [55–57]. Together, these have facilitated fine detail-mapping of DNA methylation and its derivatives (e.g. 5^hm^C), captured histone modifications in single cells, and they have significantly contributed to chromatin accessibility studies, such as chromosome conformation capture (3C) technologies [58]. These are important advances as they are beginning to allow us to understand higher-order regulation of gene expression and how it is linked to cellular plasticity and diversity.

Nevertheless, a significant upcoming challenge in livestock breeding is to track epigenetic information that changes from one generation to another. However, it has been known for some time that a significant proportion of the phenotypic variance is explained by paternally imprinted loci where one allele’s expression differs from the other because expression depends on the parent from whom it was inherited [59–61]. Nowadays, the current genetic improvement scheme in livestock assumes that the expression of desirable traits is dependent of parental origin [62]. These traits show a complex inheritance, which is the result of multiple combined genetic and environmental factors. According to animal breeding theory, most of the traits are affected by a large number of genes but each individual gene contributes only very little to the overall phenotypic variance of the trait. Importantly, individual gene effects, or more precisely effects of chromosomal regions, are detectable in quantitative traits [63]. Some of these, referred to as quantitative trait loci (QTLs), show parent-of-origin-specific effects and comprise imprinted loci [64]. This asymmetric allelic expression is established via epigenetic mechanisms during development of germ cells into sperm or egg—see below [65]. An imprinted gene is in effect heterozygotic, making it more vulnerable to negative mutational effects that are often connected to disease. Hence, a single mutation can have dramatic phenotypic consequences [66].

Until recently it was thought that dosage compensation does not occur in birds. However, we now know that many Z-linked genes in chicken are indeed dosage-compensated [67]. The process does not involve sex chromosome inactivation typical of mammals but rather some unknown mechanism [68]. In poultry, it has been suggested that QTLs for economically important traits, such as egg weight, age at first egg, feed intake, egg quality and body weight with parent-of-origin-specific expression, could be the result of genomic imprinting, which is often assumed to be unique to mammals [68]. However, differentially methylated alleles in the chicken genome have yet to be identified experimentally [69]. Furthermore, genes such as *Mpr*/*Igf2r* and *Igf2* that are imprinted in mammals are all expressed biallelically in birds [13].

In parthenogenesis, growth and development of embryos occur without fertilization. Consequently, expression of imprinted genes is severely affected. Developmental studies of parthenogenesis in sheep foetuses identified effects on growth and subsequently death [3, 70], and the unbalanced expression of imprinted genes is thought to be involved in these severe effects. Many imprinted human and mouse genes are also imprinted in sheep [3, 71, 72] (Table 1). Moreover, many imprinted genes have been detected across numerous species including cow, sheep, dog, pig, rabbit, chicken, opossum, lab opossum, human, mouse, rat and wallaby (http://www.geneimprint.com/site/genes-by-species) (Table 1). The current number of confirmed imprinted genes in livestock (cow, sheep, dog, pig, chicken, Table 1) is about 60 (26.3 %), and most of them are found in pig and cow. Importantly, there is an increasing interest in the role of certain imprinted genes, such as *IGF2*, in livestock because it is thought to play a role in the variation of complex production traits, such as muscle mass and fat deposition in pigs as well as meat and milk production in beef and dairy cattle [73].

**Table 1:** List of imprinted genes by species (adapted from http://www.geneimprint.com/site/genes-by-species)

Organism	Imprinted	Paternally expressed	Maternally expressed	Other^a^
Cow	20	12	8	–
Sheep	16	6	8	2 (1× isoform dependent, 1× unknown)
Dog	1	–	1	–
Pig	22	14	6	2 (1× tissue dependent, 1× biallelic)
Rabbit	1	1	–	–
Chicken	–	–	–	–
Opossum	2	1	1	–
Lab Opossum	6	2	4	–
Human	97	61	29	7 (4× isoform dependent, 2× random, 1× unknown)
Mouse	124	50	62	12 (5× isoform dependent, 7× unknown)
Rat	6	3	3	–
Wallaby	5	4	1	–

^a^Other: non-paternal or maternal form of allele expression in the zygote

Importantly, implementation of improved breeding programs, where imprinting is taken into account, will require changes to the current standard breeding programs. This will allow to include variables, such as different breeding values for males and females, dominance deviations and additive genetic variances [63]. Continuing the theme of this section, ‘challenges in epigenetics for livestock breeding’, we will highlight later in this review further challenges, as well as potential hurdles, to using epigenetic knowledge in breeding programmes and livestock science in general. For further discussions on these issues, the reader is referred to the later sections entitled (1) non-Mendelian inheritance in genetic improvement and the potential uses of epigenetics in animal breeding, (2) epigenetics in livestock immunity and (3) prospects of epigenetic therapies in livestock.

## DNA methylation in development

In order to understand how epigenetic processes may facilitate heritable transmission of information, we need to appreciate the changes to the epigenome that occur during germ cell formation and early organismal development. Additionally, we can learn much from animal cloning experiments that have recently become popular. In this section, we consider these issues with respect to epigenetic processes.

### Germ cells

The relative abundance of methylated DNA within germ cells commonly varies between oocytes and sperm. For example, methylation of satellite DNA in the pig exhibits extreme hypermethylation in sperm nuclei compared to oocyte nuclei [74]. Moreover, the relative ratio of DNA methylation in sperm to oocytes also varies between species [37, 75].

The level of gamete DNA methylation can also be dramatically different to that within somatic tissues of the same organism. For example, satellite fragments within bovine sperm genomic DNA are completely unmethylated yet they are fully methylated in thymus genomic DNA [37, 76]. Similarly, gamete-specific methylation patterns observed in mouse [77–81] are satellite DNA regions that are considerably undermethylated in sperm. Furthermore, dramatic loss of cytosine methylation from the male pronucleus occurs in mouse, pig and cow [37].

Parental genomes are highly methylated throughout preimplantation development in sheep and rabbit embryos with equally high methylation levels. In mouse, the maternal genome undergoes passive DNA demethylation throughout several rounds of DNA replication, whereas the paternal genome undergoes active demethylation prior to DNA replication in the zygote [82–84]. Indeed, the paternal genome is apparently completely demethylated in this process even though the mature murine sperm genome possesses an overall CpG methylation level of 80–90 %, the highest global DNA methylation level of any cell in the mouse [85].

In contrast to the mouse and cow [37], there is no passive demethylation throughout sheep preimplantation development, but rather an apparent increase between the 8-cell and morula stages. At the blastocyst stage, demethylation becomes visible in the sheep trophectoderm (TE), whereas the cells of the inner cell mass (ICM) remain methylated. From this, it can be concluded that demethylation of the paternal genome is not an obligate requirement for early mammalian development [37]. In other organisms, such as zebrafish, paternal DNA stably maintains the sperm DNA methylome after fertilization until the midblastula (MBT) stage [86].

Intriguingly, mouse sperm DNA injected into sheep oocytes is significantly demethylated, whereas mouse sperm DNA injected into murine oocytes is only partially demethylated. Also ram sperm DNA, which is not demethylated in sheep oocytes, is partially demethylated in bovine oocytes [81]. Analyses such as these suggest that the DNA demethylating activity is dependent upon both ooplasm- and sperm-specific factors. Thus, the demethylating activity of ooplasm differs between species, being the highest in mouse, medium in bovine and low in sheep (and rabbit) oocytes. Hence, the degree of sperm and oocyte demethylation activity differs among species.

### Embryo development

A fundamental requirement for transmission of epigenetic information to the next generation is that it survives the genome-wide reprogramming of DNA methylation during early development in mammals. This leads to differences between methylation patterns in germs cells and those in embryonic cells because some genomic regions, for example those containing imprinted genes, escape reprogramming.

**Fig. 2:**
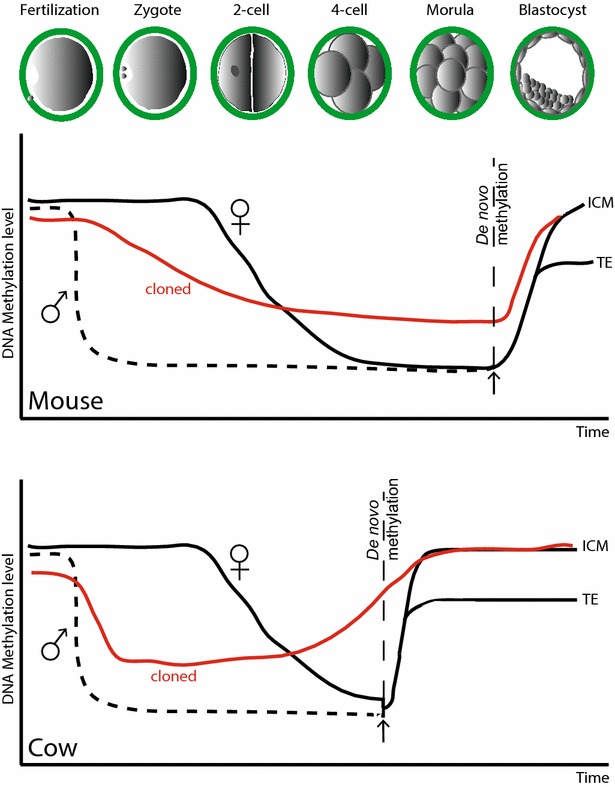
DNA methylation profiles during early preimplantation development and in clones. The paternal (*black dashed line*) and maternal (*black solid line*) genome DNA methylation profiles through preimplantation development are shown for mouse (*top panel*) and cow (*bottom panel*). Very soon after fertilization the paternal genome is demethylated in a replication-independent genome-wide manner. In contrast, demethylation of the maternal genome occurs later. In cloned embryos (*red line*), moderate demethylation occurs by the blastocyst stage, after nuclear transfer and before de novo methylation, with hypermethylation of the TE. In cattle there is active and passive demethylation followed by de novo methylation at the 8- to 16-cell stage. In cloned embryos, de novo methylation occurs at the 4-cell stage and TE is hypermethylated. *ICM* inner cell mass—cells that reside within the trophoblast and consist of pluripotent cells that give rise to the embryo. *TE* trophoblastic cells—cells that surround the blastocyst cavity and which give rise to the placenta (adapted from [83])

During embryonic development, gamete methylation marks are erased (i.e. demethylated) and replaced by embryonic marks that are important for development and appropriate cell potency. Remethylation, or rather de novo methylation, establishes the basic somatic methylation pattern around the time of implantation (Fig. 2). In bovine embryos, remethylation occurs at the 8- to 16-cell stage. In mouse blastocysts, remethylation occurs at the same 8- to 16-cell stage but here it is restricted to the cells that will form the ICM. This is because de novo methylation completes the reprogramming of DNA methylation in the mouse ICM during preimplantation development, but it does not do so in the trophectoderm at the blastocyst stage. Hence, the latter is virtually devoid of DNA methylation at this stage of development [81]. This highlights the fact that dynamic changes in DNA methylation in embryonic cells vary between species [33, 75, 80, 87, 88]. There is genome-wide DNA demethylation in mammals [89], although it is currently believed that paternal DNA is actively demethylated whereas maternal DNA is passively demethylated [79, 90]. Furthermore, in the developing mouse, cells of the ICM selectively remethylate during the morula stage (Fig. 2), while in sheep there is selective demethylation in the trophectoderm cells compared to the ICM [37], as sheep embryos do not undergo dramatic genome-wide demethylation [91]. Indeed, this observation in sheep brings into question the role of DNA methylation in preimplantation development. Furthermore, mice deficient in de novo DNMTs display no phenotype prior to implantation [92].

Differences between species are also evident when one considers histone modifications during early preimplantation development. For instance, in mouse, following fertilization, the paternal genome takes on H3K27me3 to form facultative-like heterochromatin before H3K9me3 is finally deposited. In humans, the paternal genome is immediately marked with H3K9me3 [93] demonstrating a striking difference between the species, although the underlying reasons are unknown.

### Cloned animals

Cloned animals are derived from a single genome of somatic DNA. Animals, including outbred strains, derived by somatic cell nuclear transfer (SCNT) frequently exhibit pathogenic alterations that can be linked to inappropriate nuclear reprogramming during early embryo development, supporting the studies for a link between epigenetics and disease. A great deal of information on the significant epigenetic features governing early embryo development has been provided by the ground-breaking work of Gurdon, Wakayama, Hochedlinger and Jaenisch, among others [94–98]. These experiments formed the basis for other studies, including nuclear transfer experiments in mammals and somatic cloning of sheep [99, 100].

Zygotic SCNT involves insertion of a somatic nucleus into an enucleated recipient egg such that it confers normal development. For this to occur, oocyte-specific factors must reprogramme the donor genome in such a way that it allows the coordinated expression, both temporally and spatially, of all necessary genes. This involves genome scale remodelling of chromatin. If successful, the accommodated nucleus is considered ‘synchronized’ with the enucleated oocyte [101]. Notably, differentiated nuclei possess an intrinsic resistance to reactivation of genes needed for early development and this reduces reprogramming efficiency compared to the efficiency using nuclei from less differentiated cells [e.g. embryonic stem cells (ESs)] [101]. One component of this inhibition is histone H3 variant H3.3 [101]. Additionally, even if reprogramming happens, memory of an active gene state is maintained within donor nuclei and this can cause interference with lineage selection in nuclear transplant derived embryos. For example, embryos derived from transplanted muscle cell nuclei overexpress muscle-related genes [94, 101]. This memory is likely to have an epigenetic basis.

Changes in DNA methylation status can have profound effects on gene expression in cloned animals, and epigenetic irregularities are believed to influence imprinted genes in particular [102–107]. Indeed, studies in cloned bovine embryos suggest that most SCNTs fail because of hypermethylation changes in the DNA of cloned preimplantational embryos [108]. Although most offspring derived from somatic cloning are normal [109], some somatic cloning protocols are associated with pathologic side effects, which appear to be due to incomplete and/or faulty epigenetic reprogramming [110]. Indeed, a general dramatic epigenetic reprogramming occurs in SCNT when the expression profile of a differentiated cell is abolished and a new embryo-specific expression profile is established, which drives embryonic and foetal development [111]. DNA methylation and histone modifications are critical for this process, with imprinted genes being particularly sensitive, and key roles have been proposed for small ncRNAs and proteins with domains that bind to methylated and non-methylated DNA [112]. Importantly, the kinetics of DNA de/methylation in cloned embryos after nuclear transfer differs from that in fertilized embryos. Also, demethylation is taking place by the blastocyst stage in mouse, before de novo methylation, and there is hypermethylation of the TE [83]. The dynamics of DNA de/methylation in normal and cloned embryos of mouse and cattle are illustrated in Fig. 2.

SCNT is currently an inefficient procedure because of the erroneous reprogramming of the donor genome [113]. An example of erroneous reprogramming is ectopic expression of *Xist*, a ncRNA essential for X chromosome inactivation. Ectopic expression of *Xist* may adversely affect gene expression in cloned embryos in a genome-wide manner, probably through complex gene networks connecting autosomal and X-linked genes that direct embryonic development [113]. If its expression could be controlled, there would be a great potential to improve mammalian SCNT efficiency and fidelity.

It has been suggested that miRNA technology and exploitation of histone demethylases, such as the H3K27 demethylase Utx, represent an important approach to redirecting the epigenetic reprogramming of somatic cells to pluripotency [111]. Using this approach, it may be possible to control *Xist* expression in cloned embryos, for example by post-transcriptionally blocking its ectopic expression. This would most likely improve the current SCNT cloning efficiency. Improving the efficiency of mammalian SCNT to a practical level (e.g. >20 % per embryos transferred) will have many potential applications in biology, regenerative medicine and agriculture [83]. However, given the limitations to the current technology of animal cloning, it seems unlikely that this procedure will be a major consideration regarding livestock breeding within the near future.

## Environmentally induced heritable traits

Historically, there have been numerous formal considerations and experimentation suggesting that different types of trait can be transmitted from one generation to the next. It was the naturalist Jean-Baptiste Lamarck who initially proposed in the eighteenth century that the environment can influence heritable changes in living cells within a generation or two [114]. Subsequently, in the nineteenth century, physiologist and physician Ivan Pavlov substantiated the Lamarckian principles with a remarkable discovery that the offspring of a mouse that had learnt to navigate a maze had inherited the acquired external memory and learnt the maze faster than their parents had done [115]. Later on, it was shown that dietary supplementation of vitamin B, a methyl donor, had a prominent effect on DNA methylation and DNA methylation induced by the B vitamins (nutrient signal, Fig. 1) facilitated the generation of healthy puppies, which were not prone to diabetes [116]. More recently, there has been accumulating evidence indicating that individuals can acquire environmentally induced epigenetic marks to form a type of transgenerational memory.

Certain environmentally induced changes in the epigenome are recorded in genomic DNA methylation patterns for up to three generations [117]. Among the classic examples, in humans, mothers who were pregnant during the food crisis in The Netherlands in 1944, also known as the ‘Hunger Winter’, had children and, crucially, grandchildren with a wide range of health disorders [66, 118]. A similar pattern has also been shown in sheep as in humans, where the diet of pregnant ewes affected the weight of their granddaughters [119]. However, the pattern of DNA methylation was not assessed in these nutritional studies.

For many health reasons, diet and nutrition in animals, including humans, have long been the focus of much research interest and this has provided insight into potential epigenetic mechanisms that may be operative. For instance, nutritional changes lead to global alterations in DNA methylation and histone modification in animals, and specific effects of a selected nutrient on DNA methylation and histone modification have been reported (Table 2).

**Table 2:** Summary of effects of selected dietary factors on DNA methylation and histone modifications in animals and in vitro systems

Nutrient/diet component	Observation relevant to DNA methylation	Refs.
Methyl donor	Maternal supplementation with methyl donors reversed the effects of maternal bisphenol-A exposure during pregnancy on methylation at the *Avy* of the offspring	[193]
Folic acid	Changes in *tp53* gene expression and DNA methylation status of intrauterine growth retarded rats were reversed by dietary folic acid supplementation	[194]
	Folate deficiency did not alter genomic DNA methylation in the liver of BALB/c mice	[195]
Selenium	Se inhibits DNMT1 activity in vitro from rat liver and Friend erythroleukaemic cells and in prostate cancer cells causes induction of *GSTP1*, *APC* and *CSR1* gene promoter demethylation and reduction in histone deacetylases (HDAC) activity leading to increased acetylation of H3K9 and gene expression	[196]
Protein	Dietary supplementation with folic acid prevented hypermethylation in imprinting control region of *IGF2* and *H19* genes with low-protein diet only	[197]
	Promoters of 204 genes were differentially methylated in murine foetal liver in response to low-protein feeding during pregnancy. The promoter of the liver X-receptor alpha was significantly hypermethylated by the protein restriction	[198]
Fatty acids	Fat exposure during development induces persistent changes in hepatic polyunsaturated fatty acid status in offspring through epigenetic regulation of fatty acid desaturase gene (*Fads2*) transcription	[122]

	Observation relevant to histone modifications	Refs.
	*Model of porcine kidney fibroblasts (PKFs)* NaBu-induced hyperacetylation up-regulates Wilms' tumor 1 (*WT1*) gene expression essential for the development of kidney fibroblasts (PKFs) suggesting histone acetylation mediation in the transcriptional modulation of *WT1* in porcine kidney cells	[199]
Butyrate		
	*Model of mouse adipose tissue* Mice heterozygous for a mutation in *Trim28* (known as KAP1 or TIF1-β), an epigenetic-modifier complex that directs a repressive chromatin state, develop liver steatosis, adipocyte hypertrophy and impaired glucose tolerance	[200]
Fat		

Recent work indicates that folate is used to regenerate methionine and hence S-adenosylmethionine, the methyl donor used by DNA methyltransferases (DNMTs) and histone methyltransferases (HMTs) [120]. In addition, a protein-deficient diet induces DNA hypermethylation in rat foetus livers [121], while fat exposure during development induces persistent changes in hepatic polyunsaturated fatty acid (PUFA) status in offspring through epigenetic regulation of the fatty acid desaturase gene (*Fads2*) [122] (Table 2). Finally, a high-concentrate corn straw diet in dairy cows induced an altered state of DNA methylation in a number of genes involved in fat and protein synthesis in the mammary tissues [123]. Observations, such as these suggest that epigenetic events help regulate nutritional signalling and the associated effects [123]. Tissue-specific DNA methylation is highly regulated at most genomic loci, resulting in little interindividual variation in epigenotype. However, it seems that specific environmental conditions are likely to affect certain methylation patterns, and these patterns can contribute to phenotypic variation between two individuals.

Genome-wide analysis in rats identified vinclozolin-induced transgenerational epimutations (i.e. DNA methylated regions) in sperm using copy number variations (CNV) [124]. Environmental induction of the epigenetic transgenerational inheritance of sperm epimutations promoted genome instability, such that genetic CNV mutations were acquired in later generations. These findings indicated that both epigenetic and genetic events were involved in the transgenerational phenotypes, representing a significant advance in our understanding of how the environment impacts disease and evolution.

So far, we have described the effects of nutrients, disease and other factors on epigenetic marks in farm animals. Only one study has examined a transgenerational epigenetic response in farm animal species in recent years [125]. Specifically, the authors investigated the effects of dietary methylating micronutrients on gene expression and DNA methylation in three generations of Large White pig. They found significant differences in gene expression between groups and in DNA methylation profiles at the promoter of the *iodotyrosine deiodinase* gene in the F2 generation [125].

Nutrition is one of the most important environmental signals affecting phenotype (Table 2). Microelements, such as zinc have been linked to epigenetic changes. For instance, a high-zinc maternal diet causes an anti-inflammatory effect via epigenetic modifications of the *A20* gene promoter in offspring chicks [126]. The effect of diet upon epigenetic parameters in larger animals has been rarely assessed. One such study, involving ruminants, investigated the effects of restricted methyl donor dietary vitamins, such as B6, vitamin B12, folate and methionine in pregnant Scottish blackface ewes [127]. The offspring of these ewes exhibited higher blood pressure, a greater tendency to obesity and insulin resistance when compared with the controls [127]. Additionally, undernutrition during late gestation in sheep causes adult hyperthyroidism associated with increased expression of genes regulating thyroid hormone synthesis and deiodination [128].

Calorie-restricted or calorie-overfed female rabbits experience significant changes in the expression levels of the deacetylase Sirtuin 1 (silent mating type information regulation 2 homologue 1; SIRT1). Specifically, rabbits in early pregnancy, fed on an obesity-inducing diet, deliver male offspring with significantly reduced SIRT1 protein expression in their livers (Triantaphyllopoulos K., unpublished observations). Importantly though, meat quality traits (e.g. carcass composition of the offspring) are not significantly affected during this period [129]. It is noteworthy that, SIRT1 is an NAD^+^-dependent deacetylase that deacetylates numerous transcription factors [130] and promotes fat mobilization, suppresses adipogenesis and regulates hepatic glucose and lipid metabolism [131]. It is likely that, these SIRT1-mediated metabolic effects involve epigenetic mechanisms as reprogramming of cellular metabolism in skeletal muscle stem cells, reduced deacetylase activity of SIRT1, elevated H4K16 acetylation and activated muscle gene transcription [132]. Thus, metabolic cues can be mechanistically translated into epigenetic modifications that regulate skeletal muscle stem cell biology.

Recently, an epigenetic switch has been identified that causes genetically identical mice to be either lean or obese [133]. Importantly, this is the first example of ‘polyphenism’ in mammals, where two or more distinct phenotypes are produced by the same genotype. TRIM28 is a large multi-domain protein that causes heterochromatin deposition and silencing by facilitating interactions between transcription factors, histone deacetylases (HDACs) and histone methyltransferases [133]. It was found that Trim28 haploinsufficiency triggers bistable epigenetic obesity in mice via reduced expression of an imprinted gene network, including *Nnat*, *Peg3* and *Cdkn1c* [133]. Notably, the same gene network was similarly altered in lean and obese human ‘identical’ twins [133].

## Animal behaviour and epigenetic variability

Epigenetic events have been linked to the causes of different psychological behaviours. For example, epigenetic regulation of *Bdnf* was found to be involved in fear extinction in mice [134]. The behavioural training provoked changes in H3 acetylation around the p1-promoter of the *Bdnf* gene, while elimination training reversed it. Consistently, stress in rats, initiated by their brief immobilization, provokes a glucocorticoid-dependent decrease in the expression of the *Bdnf* gene, associated with histone acetylation changes within the promoter region [135].

An important example of inherited epigenetic variation is provided from studies of maternal separation in young mice. This not only alters the DNA methylation profile in the germline of the affected males, and their behavioural response to adverse environments, but it is also associated with modified brain gene expression in their offspring, even when these are raised under normal conditions [136]. Furthermore, transgenerational epigenetically mediated changes in behaviour also occur in chickens as a result of both chronic stress [137] and brief periods of early social isolation [138].

Another factor associated with epigenetic modifications and subsequent behavioural effects is environmental enrichment whereby furnishing and supplying animal cages with substrates, environmental ‘enrichment’ and nesting material allows the wider expression of animals’ natural behaviour, from neuronal development to stress resistance [139]. Notably, environmental enrichment and nesting material recover inadequate and poor learning behaviour and improve long-term memories and stress resistance, even when significant neuronal loss and brain atrophy has already occurred [139]. This effect is highly correlated with chromatin modifications, especially histone acetylation, and HDAC inhibition induced dendrite growth and synapse formation [140]. This is an important finding with several implications in animal experimentation, husbandry and animal welfare.

Early childhood hardship (nursery-rearing) produces an array of behavioural, physiological and neurobiological deficits in non-human primate models that parallel those identified in human studies of early hardship [141, 142]. For example, a randomized differential rearing experiment investigated maternal versus surrogate-peer rearing in rhesus macaques. It was found that offspring exhibited mother-dependent differential DNA methylation patterns in early adulthood, leading to differential DNA methylation in both prefrontal cortex and immune cells [143]. Thus, quality of maternal care has a long-lasting impact on offspring, but the mechanisms involved in the biologically embedded responses to early social life environment are still unclear.

## Stress factors and epigenetic responses

It is clear that stress imparts epigenetic and transgenerational effects on animal behaviour. Stress is a very broad term, and its meaning differs depending on the context within which it is used. It was recently suggested that the term stress should be limited to circumstances where an animal has exceeded the natural regulatory capacity, leading to activation of the hypothalamic–pituitary–adrenal (HPA) axis and the sympathetic nervous system [144].

Morgan and Bale exposed pregnant mice to stress during the first week of pregnancy and then studied the F2 offspring of the males born after this embryonic exposure [145]. By examining gene expression patterns during the perinatal sensitive period, it was observed that males were dysmasculinized morphologically, physiologically and behaviourally. The dysmasculinized males differentially expressed certain microRNAs in their brains in a profile that closely resembled expression patterns in control females [145]. Interestingly, infants that are prenatally exposed to maternal depression or anxious mood exhibit increased DNA methylation of the glucocorticoid receptor (*GR*) gene, which is associated with a heightened cortisol response to a mild stressor [146]. Such programming effects may transmit to subsequent generations, predisposing offspring to disease [145].

Different mechanisms have been employed to provoke experimentally induced stress effects, such as maternal isolation or various kinds of chemical exposure [147, 148]. Experiments in mammals have shown that the hippocampus is an important site of negative feedback of the HPA axis, while prolonged stress in mammals is associated with a reduction in binding of both GR and mineralocorticoid receptor (MR) in the hippocampus [149]. Some ‘stressor’ challenges may have long-term positive effects and adaptive results on organisms, being significant mediators of phenotypic plasticity and playing an important role in allowing animals to adjust to changing environments.

Most of the effects and gene expression changes discussed above presumably involve changes in the epigenetic status of the relevant genes, though this needs to be formally tested. Nevertheless, the differences in early environmental conditions experienced by animals could explain much of the intraspecific variation in adult phenotype and organism fitness, which may in turn be attributed to epigenetic variation [147].

## Non-Mendelian inheritance in genetic improvement and the potential uses of epigenetics in animal breeding

Livestock breeding currently takes advantage of important molecular data collected from genotyping polymorphisms in DNA. If an inherited abnormality or phenotypic characteristic (trait) is caused by an epimutation then it would not be possible to find the cause within the DNA sequence data.

Although causal mutations involving inherited epimutations have been studied in certain animal models (inbred mouse strains), the success in finding them in livestock is currently low [150]. Despite the hurdles associated with livestock studies, distinguishing epigenetic effects (heritable or not) would provide a great benefit to animal science, husbandry, etc., resulting in an improved accuracy of prediction of breeding values [151]. Single nucleotide polymorphisms (SNPs) and stable epimutations under linkage disequilibrium (LD), could be accounted for in the same way that LD is estimated for the DNA variations alone [150]. Presumably, the genomic selection would still work even if part of the total phenotypic variance (see next section) is only contributed to by stable epimutations. In our opinion, the following issues should be taken into account when considering prediction of future phenotypes using epigenetic data in livestock.

It is not known how, significantly, epimutations contribute to genetic variance in animal breeding. Furthermore, whole genome methylation screening for SNPs and epimutations affecting various inheritable traits is very expensive, at current costing, and many animals will have to be evaluated. We envisage that genome-wide association studies (GWAS) [152] and epigenome-wide association studies (EWAS) could enormously accelerate epigenome screening in the future. Notably, a recent report indicated that human methylation BeadChip arrays may be useful for DNA methylation profiling in non-human genomic DNA samples [153]. Further improvements in this kind of technology will hopefully allow us to address issues, such as these, in the near future.

At present, we cannot accurately predict environmental variables that affect offspring. Phenotypic variance is certainly linked to genetic factors. However, it is highly likely that it is also affected by environmental and epigenetic factors. The effects of the latter are of unknown magnitude, and therefore, there is an unknown variable within the heritability equation (that predicts offspring phenotype) which is related to the environmental variance, V_E_ [154]. The environment that organisms are raised in, as well as the environment they are measured in, will be ‘recorded’ within their epigenomes, and this will ultimately affect phenotypic variance in a way that is not described by genetic variance alone. Interestingly, environment and environmental stressors are important to understanding evolutionary forces in natural populations [155].

In this review, we have implied that there is an unexplained phenotypic variation that is not due to DNA sequence information. This can be seen, for example, in cattle where only 32–80 % of the additive genetic variance can be explained by genetic variation (SNPs, substitutions, etc.) [156]. Thus, there is a ‘missing heritability’ component [157]. To account for the combined genetic and epigenetic effect, novel statistical procedures are being devised to allow the researcher to distinguish genetic from epigenetic variance [158]. For example, there are methods now to estimate epigenetic contribution to covariance between relatives [159] and we can begin to analyse the epigenetic variation [160].

The contribution of the paternal effects in phenotypic variance has also to be taken into account. In this regard, much attention is currently being focused onto *trans* effects, which involve factors existing is the sperm such as proteins and RNAs [150]. Equally important though, are the effects due to parent-of-origin inheritance (e.g. imprinted genes) that cause unbalanced gene expression (as described earlier).

It is conceivable that an epigenomic ‘code’ exists, which if deciphered may allow us to accurately predict the future phenotype. However, we are a long way from being able to achieve this. Working towards this goal, we will need to significantly improve genome- and epigenome-wide mapping and sequencing technologies and make them much more affordable. This will involve improvements to genome-wide next-generation sequencing (NGS), quantitative proteomics, computers and computational analyses [55, 159, 161]. Importantly, epigenetic modifications, such as DNA methylation, are only part of the epigenetic ‘code’ [162]. This highlights the vast range of non-genetic events that we will need to analyse and consider, if we are going to be able to accurately interpret and predict phenotype.

## Epigenetics in livestock immunity

With respect to farm animals, it is of vital importance to breed animals with a robust immune system. The quality of the immune system is portrayed in the diversity of the general characteristics including animal well-being, farm profit, milk quality and disease rates. Therefore, the optimal strategy for a farm is to invest in livestock that is superior in terms of robustness of the immune system. Ample evidence exists for selection breeding for immune responsiveness in rodents, poultry, pigs and cattle, where high responders (H-responders) can positively influence resistance to an infectious disease compared to average (A) or low (L)—immune responses (AIR or LIR, respectively) [163]. This evidence is based on differentially expressed genes between the H and L responders, including immune response transcription factors, cytokines, histocompatibility and T cell receptor genes [164]. Thus, considerable emphasis should be placed upon the breeding of dairy cows and other farm animals for enhanced immune responses. Of particular significance and importance is intrinsic resistance against life-threatening diseases in livestock (e.g. paratuberculosis in ruminants) that has the potential to affect humans following consumption of infected meat.

### Epigenetic mechanisms in animal immunity

*Tbx21* and *Gata3* genes encode the T-helper 1 and T-helper 2 cell lineage-specifying transcription factors T-bet and Gata3, respectively [165, 166]. When expressed, the relevant gene promoter contains the active gene mark H3K4me3, but when repressed the promoter contains the repressive histone mark H3K27me3 [167]. It is believed that lineage precursors maintain these gene promoters in a ‘bivalent’ state with both H3K4me3 and H3K27me3 present. In this way, the associated genes are prepared for rapid activation or silencing, depending upon which particular differentiation pathway is adopted [167]. Importantly though, the decision to adopt a particular CD4+ T cell differentiation path is determined not simply by the epigenetic states of cytokine gene loci but also the epigenetic states of the entire set of genes (and signals) associated with these lineages [167, 168]. In fact, there is an intrinsic memory to this process because CD4+ helper T cells clonally expand and adopt multiple fates yet maintain their defining epigenetic signature throughout the entire process [167].

Dexamethasone is an immunosuppressor used to simulate corticosteroid effects around parturition. Its administration to dairy cows induces epigenetic effects at the *IFN*-*γ* and *IL*-*4* cytokine promoters, with increased DNA methylation at the *IFN*-*γ* (+18 %) gene and decreased methylation at the *IL4* (−31 %) gene [169]. Moreover, the inverse methylation patterns observed at these two cytokine genes have also been reported in other species, which is consistent with their opposing regulatory functions [170].

### Microbial immunity

Genes such as the Toll-like receptor gene have been highlighted as potential DNA methylation targets of polymorphism-dependent sensitivity to MAP (*Mycobacterium avium ssp. paratuberculosis*) infection in cattle [171]. Recently, we identified polymorphisms in the 3′-UTR of the goat *SLC11A1* solute carrier gene that regulate *SLC11A1* gene expression and the animal’s overall sensitivity to MAP infection [172]. The polymorphisms are predicted to disrupt an miRNA target sequence within the *SLC11A1* mRNA and hence post-transcriptional gene silencing. However, further studies are required to shed light upon the underlying epigenetic mechanisms, such as DNA methylation, at this locus that are linked to MAP infection.

### Infectious protein forms

Another system of epigenetic control involves ‘conformational’ states of disease-causing proteins called prions that are responsible for diseases, such as scrapie in sheep. Alarmingly, there are continuous alerts from the scientific community and social media warning of prion disease expansion and scrapie breaching the species barrier [173].

Prion disease affects many animal species, especially the ruminants. Since the 1980s, over 181,000 cases have been reported throughout Europe, but the incidence rate now has dramatically declined [174]. Nevertheless, infected livestock represent a significant potential hazard as it could provide a transmission route for the prions to infect people. Recently, misfolded prion protein entities have been created with recombinant DNA technology using recombinant mouse prion protein (PrP), providing strong evidence in support of the protein-only hypothesis, where protein alone is sufficient to transmit disease [175]. However, the exact role of RNA–protein and possible chromatin interactions are yet to be elucidated.

### Tumourigenesis in livestock

A potential link exists between DNA methylation status and epigenomic profiles on one hand and tumourigenesis on the other, but few cases have so far been examined in livestock. However, the DNA methylation status of several genes has been linked to the resistance to Marek’s disease (MD), a chicken lymphoma [47]. Hypermethylation of the *ALVE* (avian leukosis virus subgroup E) region of DNA was identified in different tissues of MD-resistant White Leghorn chicken lines. The presence of the DNA methylation inversely correlated with *ALVE* mRNA levels when compared to control chicken lines [176]. The authors suggested that the hypermethylation pattern in the *ALVE* region may impart resistance against ALV-induced tumours in chicken.

Another example of a potential link between DNA methylation and tumourigenesis comes from studies of the *BDNF* gene that was shown to be involved in tumour progression in the MD-resistant chicken. High levels of DNA methylation were identified in a *cis*-acting element of the *BDNF1* gene which is linked to the expression of a particular alternate splice form of BDNF1 RNA. High levels of DNA methylation were correlated with low expression levels of the *BDNF* RNA isoform. Importantly, this isoform has been putatively linked to tumour progression [177].

## Prospects of epigenetic therapies in livestock

Changes in the epigenome have the potential to alter any gene expression programme and consequently be linked to many altered physiological states, and in many cases diseases such as diabetes. Crucially though, and unlike DNA mutations, it should be more straightforward to reverse changes within a ‘diseased’ epigenome back to that of a non-diseased cell. This has obvious implications for human medicine as well as livestock care.

The amount of DNA methylation in a cell is tightly regulated, and many studies have observed changes in the levels in cancer cells (and in cells as we age). Many processes affect DNA methylation including (1) ageing; there is a general tendency for the genome to become hypomethylated, in contrast to certain CpG islands which become hypermethylated, a situation reminiscent of that found in many cancer cells [178], (2) diet; mammals fed a low folate and methionine diet undergo altered genomic DNA methylation associated with cancer [179] and (3) heavy metals; they affect DNA methylation linked to carcinogenesis [180–183]. The case of heavy metal exposure is particularly relevant to livestock and other animal farming since the main source of these carcinogens is food, water and contaminated air.

Global changes in histone acetylation levels are also observed in malignancies, and there are numerous examples of coding mutations (e.g. *p300*/*CBP*) and recurrent chromosomal translocations (e.g. *MLL*-*CBP*) involving histone acetyltransferases [184]. The expression levels of various HDACs (Fig. 1) are altered in certain cancers; however, coding mutations are very rare [184]. Histone methylations, as well as other modifications, are also similarly linked to cancer [184]. Given the connection between aberrant epigenetic status and diseases, such as cancer, it is not surprising that much effort has been invested developing small molecules (as drugs) that target the epigenetic machinery. Broadly speaking these are divided into two classes of compounds: (1) those that target epigenetic enzymes and (2) those that target ‘readers’ of epigenetic modifications. The majority of drugs, currently approved for clinical use, target DNA methylation and histone acetylation levels by inhibiting the DNMTs and HDACs [54].

Inhibiting enzymes, such as DNA methyltransferases by drugs such as azacitidine and decitabine has shown good results against the myelodysplastic syndromes (MDS). Vorinostat, a potent HDAC inhibitor, has been passed by the FDA for clinical use in patients with cutaneous T cell lymphoma where it shows promising results. As we identify new epigenetic enzymes, it seems certain that the repertoire of drugs regulating their activity will increase and their use in animals is a logical extension to their deployment in humans.

Chromatin ‘readers’ are proteins with specialized domains that selectively recognize and bind to modifications on specific histones. An example is, bromodomain-containing protein 4 (BRD4) that contains two tandem bromodomains, termed BD1 and BD2, that bind to acetylated lysines within histone H4 [185]. Numerous reports have validated BRD4 as a good target for therapeutic intervention and this lead to development of inhibitors, which bind to the bromodomains of BRD4, thereby preventing chromatin association and transcriptional activity. These intelligently designed inhibitors have already shown good efficacy against cancers, such as MLL-translocated acute myeloid leukaemias [186].

In addition to anti-cancer activity, epigenetic inhibitors possess other interesting characteristics. For instance, BRD4 inhibitors effectively suppress murine cardiomyocyte hypertrophy, in vitro and pathological cardiac remodelling, in vivo [187]. In fact, the spectrum of physiological responses achieved with a single inhibitor highlights how useful this approach is going to be in human medicine and more widely in animal welfare.

## Conclusions

In this review, we have considered several environmental cues in development and adult life, with emphasis on diet, stress and the disease–immunity relationship, all of which are connected to epigenetic events that alter the phenotype. During the period of developmental plasticity, epigenetic factors are at the interface between stimuli and acquired long-lasting molecular, cellular and behavioural phenotypes.

Epigenetic memory is important in ensuring sustainable viable offspring in mammals, but it is also a key player in establishing a diseased state [188]. A better understanding of DNA methylation and other epigenetic modifications will help us to link molecular, cellular, physiological and immune responses that control disease resistance. Certainly, DNA methylation is inextricably connected with the memory process, and the lack of imprinting is connected to cancer sensitivity. Interestingly, DNA methylation patterns are transmitted from maternal to daughter chromatids during mitosis and the degree of fidelity in this transmission is about three orders of magnitude lower than that of DNA sequence [189]. Stochastic epigenetic instability is more common than environment-induced changes and it can generate significant epigenetic variability over time across cells, despite the DNA sequence identity of the cells [190]. Thus, stochastic changes to the epigenome helps explain why phenotypic variation appearing in populations of inbred animals is as large as that in outbred animals, despite the fact that are both being raised in controlled environments [191].

The integration of gene expression profiles with genome-wide mapping studies of DNA methylation and histone marks is rapidly becoming a reference tool in animal and human research. It is now crucial that this methodology is fully imported and implemented into livestock guidance and consolidation programmes to ensure the most optimal diet and nutrient regimes. Also, the recognition and appreciation of the importance of transgenerational epigenetic inheritance for animal breeding purposes [192] will promote more research into established transgenerational epigenetic effects and their applications towards livestock production. This combined approach will help us to generate a unified genetic/epigenetic and multidimensional informative matrix that integrates all knowledge relevant to animal practice. This will mean that farms could use epigenetic information to reduce disease incidence and mortality rate, as well as to reduce the use of antibiotics in animal production. Indeed, the future is promising and the concept of ‘epigenetic’ drug design has already become a reality with a proof-of-principle for this approach being provided by the specific inhibitors that target chromatin-binding domains [186]. This is just the beginning of a novel therapeutic approach—one approach that should be widely applicable to many different animal diseases and pathologies.

It has taken a long time to fully accept that phenotypic complexity is not just a simple matter of Mendelian genetics, and we realize that our comprehension of the mechanisms involved is far from complete. The goal now is to expedite research in epigenetic processes to provide a better understanding of the underlying mechanisms governing full phenotypic determination and overall animal health.

## Abbreviations

3C: chromosome conformation capture
5mC: 5-methylcytosine
5hmC: 5-hydroxymethylcytosine
ATP: adenosine triphosphate
CpG: a cytosine followed by a guanine nucleotide
AIR: average immune responses
BRD4: bromodomain-containing protein 4
CD4+: cluster of differentiation 4+
DMR: differentially methylated region
DNA: deoxyribonucleic acid
DNMT: DNA methyltransferase
CNV: copy number variations
ESs: embryonic stem cells
EWAS: epigenome-wide association studies
GR: glucocorticoid receptor
GWAS: genome-wide association studies
HDAC: histone deacetylase
H3K4me2: dimethylation of histone H3 lysine 4
H3K9me3: trimethylation of H3K9
HMTs: histone methyltransferases
HPA: hypothalamic–pituitary–adrenal
ICM: inner cell mass
LD: linkage disequilibrium
LIR: low immune responses
lncRNAs: long ncRNAs
MBD: methyl-CpG-binding domain
MBT: midblastula transition
MLL: myeloid/lymphoid or mixed-lineage leukaemia
MR: mineralocorticoid receptor
NAD^+^: nicotinamide adenine dinucleotide
ncRNAs: non-coding RNAs
NGS: next-generation sequencing
piRNAs: piwi RNAs
PrP: prion protein PrP
PTMs: post-translational modifications
PUFA: polyunsaturated fatty acid
QTLs: quantitative trait loci
RNA: ribonucleic acid
miRNA: microRNA
SCNT: somatic cell nuclear transfer
SIRT1: silent mating type information regulation 2 homologue 1
siRNA: small interfering RNA
SNPs: single nucleotide polymorphisms
TE: trophectoderm
Tet: ten eleven translocation
TSS: transcriptional start site
UTR: untranslated region
